# An open-label multiyear study of sargramostim-treated Parkinson’s disease patients examining drug safety, tolerability, and immune biomarkers from limited case numbers

**DOI:** 10.1186/s40035-023-00361-1

**Published:** 2023-05-22

**Authors:** Katherine E. Olson, Mai M. Abdelmoaty, Krista L. Namminga, Yaman Lu, Helen Obaro, Pamela Santamaria, R. Lee Mosley, Howard E. Gendelman

**Affiliations:** 1 Department of Pharmacology and Experimental Neuroscience, Center for Neurodegenerative Disorders, University of Nebraska Medical Center, Omaha, NE 68198 USA; 2 Great Plains Center for Clinical and Translational Research, Nebraska Medicine, Omaha, NE USA; 3 Neurology Consultants of Nebraska, PC and Nebraska Medicine, Omaha, NE USA

**Keywords:** Parkinson’s disease, Granulocyte–macrophage colony-stimulating factor, Unified Parkinson’s Disease Rating Scale, Regulatory T cells, Neuroprotection, Sargramostim therapy

## Abstract

**Background:**

The clinical utility and safety of sargramostim has previously been reported in cancer, acute radiation syndrome, autoimmune disease, inflammatory conditions, and Alzheimer’s disease. The safety, tolerability, and mechanisms of action in Parkinson’s disease (PD) during extended use has not been evaluated.

**Methods:**

As a primary goal, safety and tolerability was assessed in five PD patients treated with sargramostim (Leukine^®^, granulocyte–macrophage colony-stimulating factor) for 33 months. Secondary goals included numbers of CD4^+^ T cells and monocytes and motor functions. Hematologic, metabolic, immune, and neurological evaluations were assessed during a 5-day on, 2-day off therapeutic regimen given at 3 μg/kg. After 2 years, drug use was discontinued for 3 months. This was then followed by an additional 6 months of treatment.

**Results:**

Sargramostim-associated adverse events included injection-site reactions, elevated total white cell counts, and bone pain. On drug, blood analyses and metabolic panels revealed no untoward side effects linked to long-term treatment. Unified Parkinson’s Disease Rating Scale scores remained stable throughout the study while regulatory T cell number and function were increased. In the initial 6 months of treatment, transcriptomic and proteomic monocyte tests demonstrated autophagy and sirtuin signaling. This finding paralleled anti-inflammatory and antioxidant activities within both the adaptive and innate immune profile arms.

**Conclusions:**

Taken together, the data affirmed long-term safety as well as immune and anti-inflammatory responses reflecting clinical stability in PD under the sargramostim treatment. Confirmation in larger patient populations is planned in a future phase II evaluation.

*Trial registration*: ClinicalTrials.gov: NCT03790670, Date of Registration: 01/02/2019, URL:https://clinicaltrials.gov/ct2/show/NCT03790670?cond=leukine+parkinson%27s&draw=2&rank=2.

**Supplementary Information:**

The online version contains supplementary material available at 10.1186/s40035-023-00361-1.

## Introduction

Globally, the prevalence of Parkinson’s disease (PD) disabilities is increasing at rates faster than any other neurodegenerative disorders; with more than 8.5 million affected worldwide. PD signs and symptoms are tremor, rigidity, limited movement, impaired balance, and poor coordination, which parallel loss of nigrostriatal dopamine and dopaminergic neurons [1]. Symptomatic control of disease is achieved by levodopa, dopamine agonists**,** and monoamine oxidase-B inhibitors [2]. Palliative therapies include modified diet and exercise, blood pressure control, and improved coordination [3]. PD is a multifactorial disease due to an interplay between genetic and environmental factors [4]. Animal models, epidemiology, neuropathology, and cellular-based research have promoted the idea that immunity and mitochondrial function play a central role in disease pathophysiology [5–7]. Both lead to deficits in bioenergetics, reactive oxygen production, and immune homeostasis. All affect the pathways of PD neuronal cell death. Lewy bodies containing aggregated and post-translationally modified alpha-synuclein (α-syn) released into the extraneuronal environment increase effector T cell (Teff) populations, which exacerbate disease outcomes [8, 9]. Our prior findings also suggest the protective and anti-inflammatory potential of regulatory T cell (Treg) populations in PD neuronal sparing [10]. Therefore, the present study focuses on peripheral aberrant innate and adaptive immune pathways that affect nigrostriatal degeneration and tests a novel therapeutic strategy to shift neurotoxic immunity into a neuroprotective response that could influence disease [11–18].

Sargramostim, recombinant human granulocyte-macrophage colony-stimulating factor, is known to affect myeloid recovery in bone marrow transplantation or chemotherapy during cancer therapy [19]. Its ability to shift proinflammatory Teff to Treg immune responses has also been demonstrated in a broad range of animal models [11, 14, 17, 20–24]. Additionally, its clinical utility has been reported for AD, PD, COVID-19, Crohn’s disease, acute radiation syndrome, and melanoma [25–32]. However, to date, sargramostim has not been evaluated for extended times nor has any immune-based biomarker been used in clinical drug evaluations in PD [28, 33]. The reported dosing regimen of 3 μg/kg with a 2-day drug holiday was chosen based on its tolerability. The primary objective of this study is to test the safety and the secondary objective is to test the effects of Sargramostim on immune profiling, CD4^+^ T cell and monocyte biomarkers, and clinical motor function.

## Materials and methods

### Study design and subject enrollment

This report served to evaluate the safety and tolerability of sargramostim (Partners Therapeutics, Inc., Lexington, MA) administered subcutaneously at a dose of 3 µg/kg for 33 months in a 5-day on and 2-day off regimen. Five subjects who met the study inclusion criteria were recruited. Patients were evaluated for 3 months to assess baseline immune, hematological, and metabolic profiles. Following baseline evaluations, subjects began to receive sargramostim therapy. The study evaluation continued for 33 months. At 2 years of continuous treatment, drug use was discontinued for 3 months. This was then followed by an additional 6 months where treatment was reinstated. One of five subjects halted study after 25 months, selecting deep brain stimulation treatment. Eligibility criteria included 35–85 years of age with PD signs and symptoms that included bradykinesia, tremor, and muscle rigidity persisting for longer than 3 years with less than stage 4 on the Hoehn and Yahr disease scale. Exclusion criteria included poor venous access, inability to undergo leukapheresis, use of a wheelchair, walker or cane, diagnosis of multiple system atrophy, corticobasal degeneration, or unilateral Parkinsonism of > 3 years. Prior head injury, stroke, brain surgery including deep brain stimulation, a family history of > 1 blood relative with PD, mental illness, cognitive impairment, autoimmune, systemic inflammatory or hematologic diseases, current treatment with neuroleptics or lithium, past treatment with sargramostim, prior immunosuppressive treatments, or known allergies to colony-stimulating factors or yeast-derived products were also exclusions.

### Ethics

The research study protocol was continuously approved by the UNMC Institutional Review Board during the entire course of study (IRB Protocol 839-18). Subjects were referred to the Clinical Research Center by their primary care physician or the study neurologist. Subjects were enrolled after informed consent was obtained by the study physician following Good Clinical Practice standards. No randomization or blinding was performed, as all study subjects were provided treatment. The trial is registered at ClinicalTrials.gov, identifier: NCT03790670.

### Procedures

The current study was a continuation of our previous year-long evaluation in which the same subjects and study protocol were followed [28]. Table 1 indicates subject demographics at the time of entry and carbidopa–levodopa therapy. Anti-parkinsonian therapies that included carbidopa–levodopa were continued during the study course. Any modifications in frequency of dosage are also listed in Table 1 and were made solely to assist in control of tremor, freezing, and reduced levodopa effects at the end of the dose interval. Anti-parkinsonian medications were allowed to be adjusted depending on the condition of the subjects during the sargramostim trial. PD subjects underwent three baseline appointments to determine initial hematologic, metabolic, and immune profiles (Additional file 1: Tables S1–S3). After baseline assessment, subjects initiated self-administration of sargramostim (Leukine, recombinant human granulocyte–macrophage colony stimulating factor [rhu GM-CSF]) at 3 μg/kg per day (5 days on, 2 days off) subcutaneously for 24 months, returning for clinical assessments every 4 weeks or 8 weeks. After 24 months, drug cessation and wash out occurred for 3 months, followed by re-introduction of sargramostim for an additional 6 months. At each clinical visit, peripheral blood samples, physical examinations, and motor assessments were completed. The study neurologist performed Unified Parkinson’s Disease Rating Scale (UPDRS) assessments in an “on” anti-parkinsonian drug state, noted observable clinical adverse events, and determined their likelihood of relationship to treatment. In between clinical visits, participants were provided an “adverse event log” that was discussed and recorded during scheduled clinical evaluations. White blood cell (WBC) counts with differentials, comprehensive blood chemistry profiles, and CD4^+^ and CD8^+^ T cell percentages and ratios were monitored for safety. Additionally, peripheral blood was stained with fluorescently-conjugated monoclonal antibodies against CD4 (FITC or AF700), CD127 (PerCP-Cy5.5), CD25 (PE), forkhead box P3 (FOXP3) (AF647), Helios (AF-488), CD152/CTLA-4 and/or iCTLA-4 (APC), CD95/FAS/Apo1 (APC), CD39 (APC), CD31 (AF647), CD27 (APC), CD45RA (AF700), CD45RO (APC), CCR7 (PE-Cy7), Integrin β7 (APC) (all from BD Biosciences, San Jose, CA), and CD49d (PE-Cy7) (BioLegend Inc., San Diego, CA), with isotype-matched antibodies serving as negative controls. Populations were gated, as previously described [28]. Fluorescent labels were examined with an LSR II flow cytometer (BD Biosciences) and analyzed using the BD FACSDiva software. Immunosuppressive function, cellular assays, and quantification of anti-sargramostim antibodies were performed as previously described [26, 28].

**Table 1:** Subject demographics

	*n*	Mean (SD)
Age (years)	5	64 (5)
Time since diagnosis (years)	5	8 (5)
UPDRS Part III Score (baseline)	5	20 (5)

	*n*	Percentage
Male sex	5	100
Caucasian race	5	100
Levodopa equivalent dose at baseline and at the end of study		
0 mg and 200 mg^a^	1	20
142.5 mg and 427.5 mg	1	20
400 mg and 800 mg	1	20
600 mg and 800 mg	1	20
800 mg and 800 mg	1	20

Demographic data taken from subjects at the time of enrollment

^a^Subject 2001 began anti-parkinsonian therapy on month 8

### Outcomes

The primary study endpoint was drug safety and tolerability assessed by complete blood counts with differential, comprehensive blood chemistry profiles, physical examination, and changes in UPDRS scores. Hematological and blood chemistry profiles were performed by a clinical diagnostics laboratory, and one neurologist performed all clinical examinations. Adverse events were recorded and scored based on event severity as mild (score, 1), moderate (2), or severe (3). Mild events caused minimal discomfort or concern and did not interfere with daily activities. Moderate events were defined as discomfort, inconvenience, or concerns ameliorated with simple therapeutic measures. Severe adverse events were defined as discomfort or incapacitation that may require prescription drug therapy, other treatments, or interventions. Events were also scored in relation to drug treatment as unrelated (score, 1), unlikely (2), possible (3), probable (4), or definitely related (5) as described [28]. No adverse events required interruption of treatment, and all safety events were evaluated and monitored by a data and safety monitoring board consisting of UNMC physicians and faculty while on study. Secondary outcomes were peripheral blood immune profiles, cellular function, cellular genomic and proteomic profiles, and presence of anti-drug antibodies over time.

### Studies of disease-linked monocyte pathways affected by sargramostim

Prior research led to insights into the potential contribution of impaired autophagy machinery to α-syn accumulation and degeneration of dopaminergic neurons in PD pathology [34]. Based on the autophagy signature observed in the monocyte proteome after 6 months of sargramostim treatment in our previous report [33], we assessed genetic links to autophagy and motor function at this treatment stage. Whole blood was collected from patient blood samples, before starting the treatment and 6 months after treatment initiation, into tubes containing ethylenediaminetetraacetic acid and monocytes were isolated by centrifugal elutriation following established protocol in our laboratories[25]. Isolated monocytes were stored in freezing medium (fetal bovine serum [FBS] with 10% dimethyl sulfoxide) and kept in liquid nitrogen until assessment of the autophagy function. After thawing the samples, viable recovery was 90%–95% of the number of cryopreserved cells (10 × 10^6^ cells/vial) and microscopic examination showed normal cellular morphology [33].

For genetic analysis, total RNA was isolated using RNeasy Mini Kit (Qiagen, Germantown, MD), and cDNA was generated utilizing RevertAid First Strand cDNA synthesis kit (Thermo Fisher Scientific, Waltham, MA) followed by amplification and quantification using RT^2^ Profiler Human Autophagy 96-well Array (Qiagen, 330231 PAHS-084Z) with RT^2^ SYBR Green ROX qPCR Mastermix (Qiagen, 330523). The qPCR cycling conditions were 95 °C for 10 min for 1 cycle, followed by 40 cycles of 95 °C for 15 s and 60 °C for 1 min using Eppendorf Mastercycler ep realplex 2S. Fold changes were determined by Qiagen’s RT^2^ profiler analysis software (version 3.5). Ingenuity Pathway Analysis (IPA, Qiagen) was used to identify the pathways affected after 6 months of treatment. The data were compared against baseline measurements before starting treatment. Functional and pathway enrichment analyses of autophagy-related genes were conducted using Cytoscape in conjunction with the Search Tool for the Retrieval of Interacting Genes/Proteins (STRING) local network cluster enrichment and the plug-in Enrichment which provides critical assessments and integration of Protein–Protein Interaction (PPI) networks based on the enriched biological processes, molecular functions, cellular components, Kyoto Encyclopedia of Genes and Genomes pathways, and Reactome pathways. Pathways and PPI networks in IPA and STRING analyses, respectively, were considered operative at a *P* value of 0.05.

In addition, autophagy regulation was measured using the Autophagy/Cytotoxicity Dual Staining Kit (Abcam, Branford, CT) following the manufacturer’s instructions. Briefly, after thawing, monocytes were cultured in 5-ml polystyrene tubes at 10^6^ cells/ml in RPMI 1640 medium without phenol red (Thermo Fisher Scientific, 11835030) with 10% FBS at 37 °C in 5% CO_2_ for 24 h, treated with 5 μM tamoxifen (a known inducer of autophagy), and incubated at 37 °C with 5% CO_2_ for 72 h. Cells cultured in the absence of tamoxifen served as controls. Cells were stained with propidium iodide (PI; a marker of cell death) for 2 min at room temperature and with the fluorescent compound monodansylcadaverine (MDC; as a probe for detection of intracellular autophagic vacuoles) for 10 min at 37 °C. Cells were washed with cell-based assay buffer after each staining. All staining procedures were performed in the dark. Cells were then suspended in cell-based assay buffer and seeded in 96-well black culture plate (2 × 10^5^ cells/well for each sample) and MDC staining intensity was detected by Spectramax M3 (Molecular Devices, San Jose, CA) using an excitation wavelength of 335 nm and an emission wavelength of 512 nm, while PI fluorescence was assessed using excitation and emission wavelengths of 536 nm and 617 nm, respectively.

### Statistical analysis

Sample size estimates of five PD subjects were determined to provide 80% power and to afford an increased score of 1.63 (32%) in baseline immune response using a two-sided Wilcoxon test assuming normal distribution. The PD immune response was measured by fluorescence-activated cell sorting (FACS) phenotypes compared against prior study results [35]. For Treg anti-proliferative function and T cell FACS results, the immune response scores from all parameters were summed and the mean immune response determined. Finally, patients were ranked based on the overall mean immune response score determined by FACS and Treg function. Statistical analysis was performed using GraphPad Prism 8.0 software (La Jolla, CA) and Statistica v13.3 (Tibco Software, Palo Alto, CA). All values are expressed as mean ± SD. Between-group differences in means were analyzed using one-way ANOVA and *post-hoc P* values for multiple comparisons with baseline were adjusted by Dunnett’s *post-hoc* test. False discovery rates were controlled at 5% using the two-stage linear step-up procedure of Benjamini, Krieger, and Yekutieli [36]. Significant differences for these studies were selected at *P* ≤ 0.05. All correlation analyses were performed using Pearson product-moment correlation coefficients, best-fit lines were determined using linear regression, *P* values were determined for *r* values.

## Results

### Demographics

Six PD subjects were screened and assessed for eligibility, with one subject excluded due to poor veinous access. Five PD subjects were enrolled and evaluated for baseline and treatment response (Table 1). All the five subjects had similar environmental exposures and were Caucasian males, 57–69 years of age with a mean of 64 years, and had been diagnosed with PD for 3–15 years with a mean of 8 years at time of entry. Four subjects began sargramostim therapy while on anti-Parkinson's medications. Modifications in dose or frequency of anti-Parkinsonian medications during the course of therapy are listed in Table 1. One subject began anti-Parkinsonian treatment at month 8 and continued a consistent dosage until drug cessation at 24 months when the subject withdrew from study.

### Safety, tolerability, and adverse event profiles

Sargramostim at 3 μg/kg for 5 days on and 2 days off was found to be safe and well-tolerated in PD subjects [26, 28]. At least one minor adverse event was recorded in each subject. The overall score for the likelihood that adverse events were drug-related reflected a less-than-possible likelihood (2.78 ± 1.02) (Table 2). Notably, 56% (112/200) of recorded adverse events were classified as unrelated or unlikely to be related to drug treatment; whereas 37.5% (75/200) were probably or definitely related to drug. The remainder (13/200, 6.5%) were scored as possibly related to drug treatment. The most commonly reported adverse events included elevated WBC counts (5/5; 100%), injection-site reactions (4/5, 80%), falls (3/5, 60%), non-infectious skin lesions (4/5, 80%), gastrointestinal events linked to nausea (3/5, 60%), neurological dyskinesias (3/5, 60%), and ophthalmological disturbances (3/5, 60%) (Table 2). Less frequently reported events included chest pain, pain in the upper torso and extremities, headache, cardiovascular issues, sleep anomalies, and neoplasms for one out of 5 subjects (20%). Secondary infections, muscle soreness, and weight loss were also reported in 2 out of 5 subjects (40%). Adverse events that were more likely associated with treatment included elevated WBC counts, injection-site reactions, and pain in extremities (Table 2). Treatment resulted in one serious adverse event and one severe adverse event. The severe adverse event involved leg cramping that was scored as possibly related to drug therapy. The serious adverse event was a thromboembolic event that was unlikely related to drug therapy. The thromboembolic event was considered as a non-related adverse event after evaluation of past history. This subject reported thrombosis in the right transverse and sigmoid sinus that was determined to be chronic and stable since 2017. An additional MRI was performed for validiation. The event was concluded to be a chronic, stable thrombosis within the right transverse sinus, sigmoid sinus, and jugular vein, and did not constitute subject withdrawal. Additionally, and as expected, sargramostim treatment significantly increased levels of WBC, including eosinophils, neutrophils, monocytes, and basophils in peripheral blood (Additional file 1: Table S1). Evaluation of blood chemistry revealed a relatively normal comprehensive metabolic profile over time (Additional file 1: Table S2). Treatment resulted in significant decreases of aspartate transaminase, total protein, and albumin levels from baseline. However, the alterations were deemed safe and non-concerning by the study neurologist. Absolute T cell profiles and ratios also remained unchanged during treatment (Additional file 1: Table S3).

**Table 2:** Incidence, severity, and relationship of adverse events to treatment

Adverse events^a,b^ for each subject	Sargramostim Phase 1b 3 μg/kg, q5d, 33 months (*n* = 5)		
	Number	Percentage	Treatment-related likelihood^c^
Any adverse event	5	100	
Any severe adverse events	1	20	
Any serious adverse events	1	20	
Adverse event leading to withdrawal	0	0	
Possible relationship to drug/placebo	5	100	
Definitive relationship to drug/placebo	3	60	

Category, Subjects reporting			Mean ± SD
1 Abnormal Laboratory	5	100	3.7 ± 0.9
2 Injection site reaction	4	80	4.4 ± 1.1
3 Chest pain or discomfort	1	20	2.0 ± 1.4
4 Pain, upper torso & extremities	1	20	4.0 ± 0.0
5 Pain, lower torso & extremities	0	0	na^d^
6 Pain, other than extremities	1	20	4.0 ± 0.0
7 Rash, other than injection site	0	0	na^d^
8 Itching, other than injection site	0	0	na^d^
9 Edema, other than injection site	0	0	na^d^
10 Shortness of breath, wheezing	0	0	na^d^
11 Headache	1	20	3.0 ± 0.0
12 Fatigue	0	0	na^d^
13 Chills, fever	0	0	na^d^
14 Infection, any	2	40	1.0 ± 0.0
15 GI tract, nausea, vomiting	3	60	1.0 ± 0.0
16 Muscle, soreness, weakness	2	40	2.3 ± 1.2
17 Equilibrium	0	0	na^d^
18 Injury, fall	3	60	1.1 ± 0.3
19 Skin, not infection	4	80	3.3 ± 1.2
20 Cardiovascular, hematological	1	20	2.0 ± 0.0
21 Neurological, psychological, dyskinesia	3	60	1.0 ± 0.0
22 Ophthalmological	3	60	1.0 ± 0.0
23 Sleep anomalies	1	20	2.0 ± 1.4
24 Neoplasms, cysts	1	20	1.0 ± 0.0
25 Weight loss	1	20	1.0 ± 0.0

	Median	Mean ± SD	
Total adverse events/subject	25.00	39.80 ± 27.73	
Total adverse events/subject per month	1.08	1.42 ± 0.99	
Severity of adverse events^c^	1.05	1.16 ± 0.20	
Likelihood of related to treatment^c^	2.89	2.78 ± 1.02	

^a^Reported adverse events since the initiation of drug

^b^More than 2 adverse events per patient may have been reported

^c^Determined by physician (1 = Unrelated, 2 = Unlikely, 3 = Possible, 4 = Probable, 5 = Definite)

^d^na = not applicable

### UPDRS scores

**Fig. 1:**
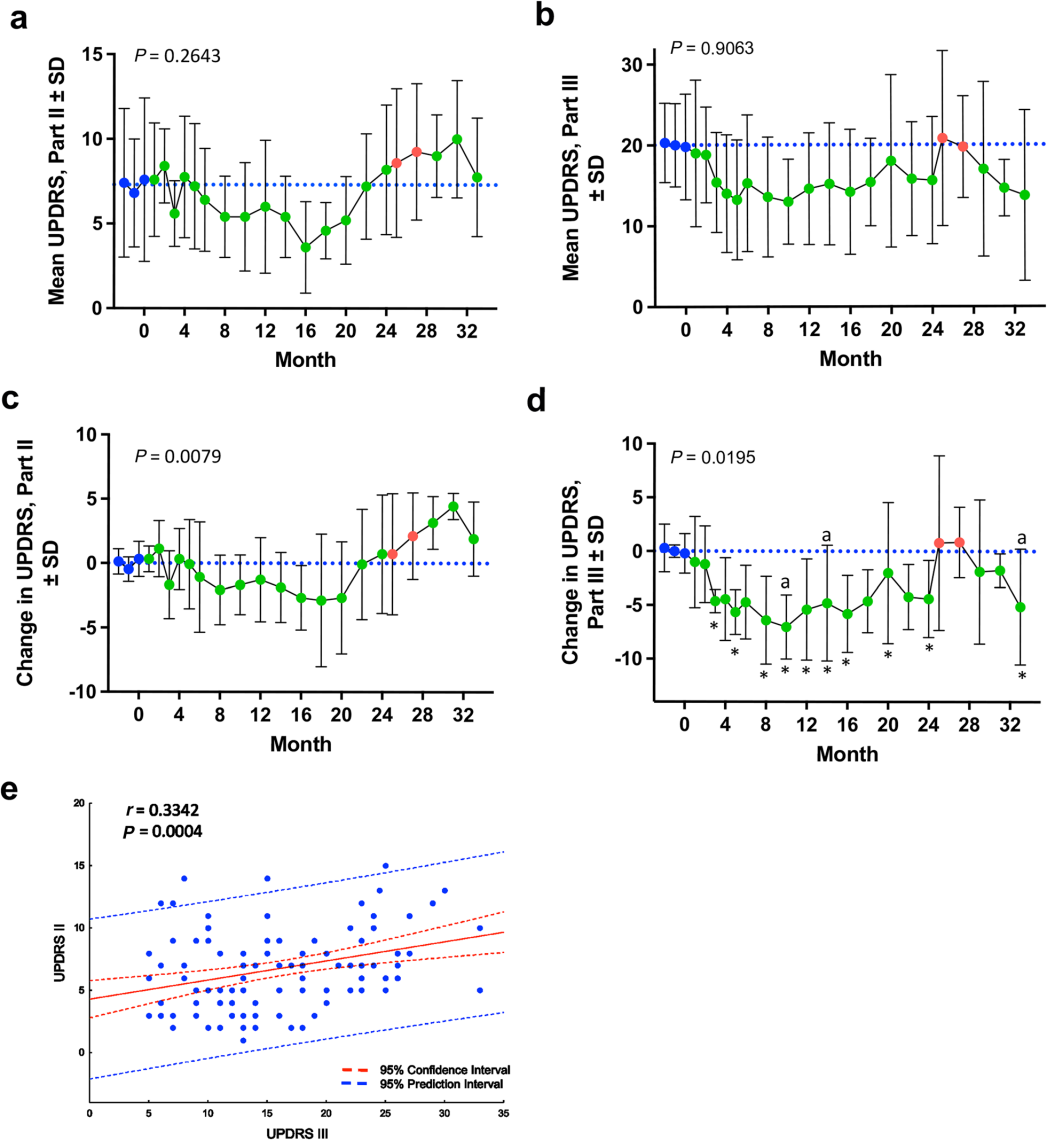
Stable UPDRS Part II and III scores are maintained during therapy. **a** UPDRS, Part II raw scores (mean ± SD) grouped by time of treatment for all subjects. **b** UPDRS Part III raw scores (mean ± SD) grouped by time of treatment for all subjects. **c** Change from baseline UPDRS Part II scores grouped by time of treatment for all subjects (mean ± SD). **d** Change from baseline UPDRS Part III scores grouped by time of treatment for all subjects (mean ± SD). Blue nodes indicate baseline evaluations. Blue dashed line indicates baseline average. Green nodes indicate “on” sargramostim treatment and red nodes indicate drug cessation. Differences in means (± SD) for each dependent variable grouped by time on treatment were determined by one-way ANOVA (*P* values annotated) and *P* values for multiple comparisons with baseline were adjusted with Dunnett's *post-hoc* test (^a^) and by false discovery rate (FDR) by the method of Benjamini, Krieger and Yekutieli [36] (*) where* P* ≤ 0.05. **e** Correlation analyses of UPDRS Part II and UPDRS Part III scores. Regression band is indicated by dashed lines that encompass the 95% confidence intervals (red) and 95% prediction values (blue). Correlation was determined using Pearson product-moment correlation coefficients, *P* values determined for correlation coefficients greater than 0.25, and best-fit lines were determined using linear regression. The Pearson *r* and *P* values are displayed on the graph. Data are depicted as scatter plots using the raw UPDRS Parts II and III scores

UPDRS Part II and III scores were monitored over 3 months prior to initiating treatment to establish the baseline motor function for assessment of disease progression. No worsening of motor function was recorded by UPDRS Part II or III (Fig. 1a, b) during the course of sargramostim treatment compared to baseline values. Sargramostim treatment resulted in non-signifcant decreases in UPDRS Part II scores, with a sustained decrease in UPDRS Part III scores by 3 months (Fig. 1c, d). The Part III scores were decreased from baseline by 4.3 ± 3.9 after 24 months of sargramostim treatment (Fig. 1d). After initiation of a 3-month drug intermission at 24 months, UPDRS Parts II and III scores returned to baseline but were not significantly elevated compared to pretreatment levels measured 24 months prior to drug intermission (Fig. 1a–d). Additionally, upon re-introduction of sargramostim, subjects experienced another decrease of UPDRS Part III motor scores below pretreatment baseline, which resulted in a significant drop below baseline levels by 6 months after re-initiation of sargramostim. Additionally, correlation analyses comparing UPDRS Parts II and III revealed a significant positive correlation of UPDRS Part II score with UPDRS Part III score (Fig. 1e). In individual subjects, the Part II scores remained stable in 4 of 5 subjects (Additional file 1: Fig. S1), while the Part III scores showed a decrease from baseline in 4 of 5 subjects and remained at baseline in one subject (Additional file 1: Fig. S2). Importantly, no subject showed UPDRS scores remaining above baseline during sargramostim treatment.

### Treg function and phenotype

**Fig. 2:**
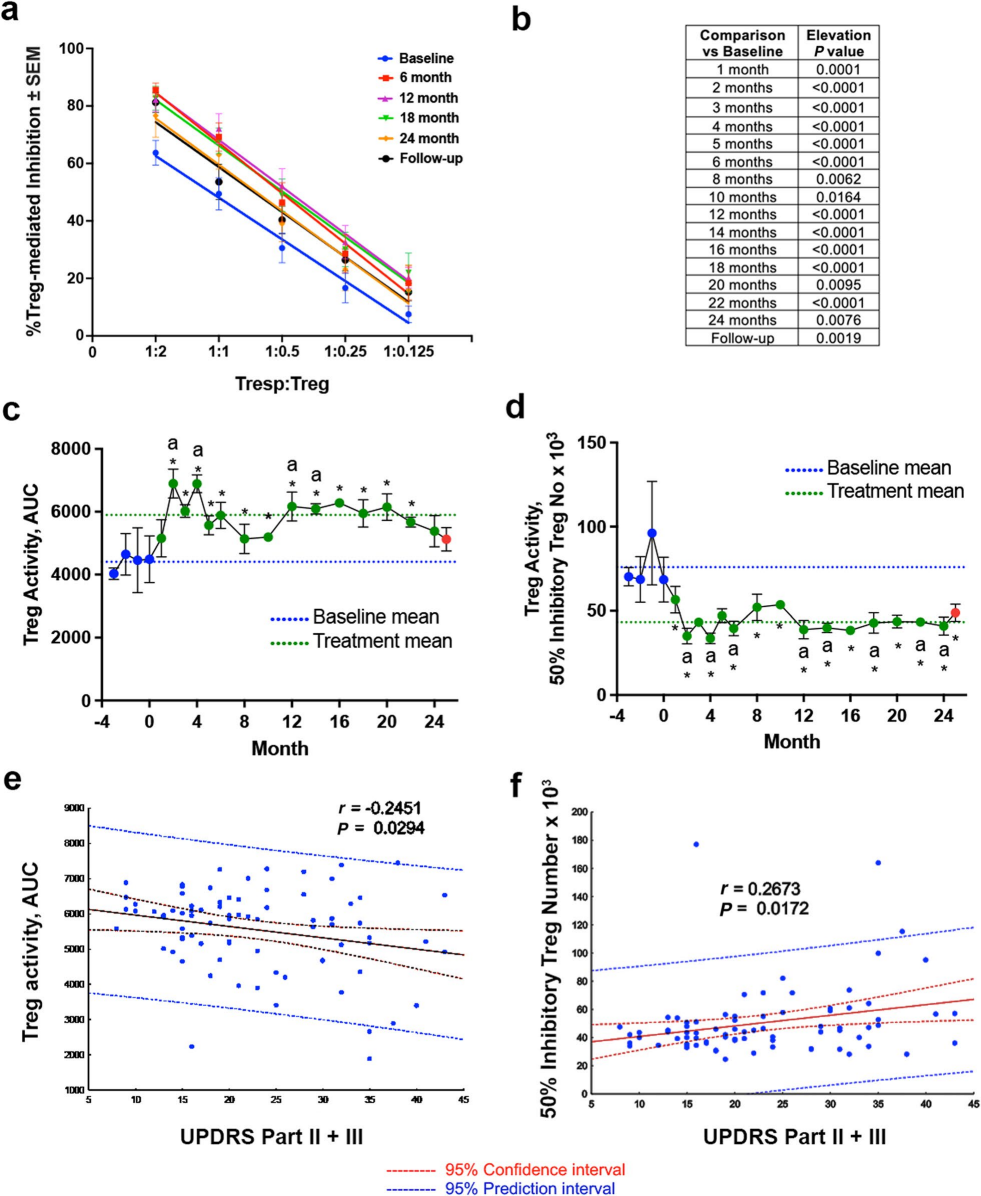
Sargramostim increases regulatory T cell function that correlates with clinical improvement. **a** Quantification of Treg-mediated suppression of Tresp (CD4^+^CD25^−^) proliferation at various Tresp:Treg ratios following every 6 months of treatment. Treg-mediated suppression is reported as percent inhibition. **b** Linear regression analysis indicates slopes with an *r*^2^ ≥ 0.67, *P* < 0.0001 for all lines and significant elevation (*P* < 0.05) from baseline at all time points. Exact *P* values for all monthly time-point elevations are listed. **c**, **d** Quantification of Treg activity as determined by the mean area under the curve (AUC) (± SEM) (**c**) and by the mean number of Tregs required for 50% inhibition (± SEM) at each sampling time (**d**). Blue nodes indicate baseline assessment, green nodes indicate sargramostim treatment, and red nodes indicate 1 month follow-up after drug cessation. The blue dashed line indicates baseline mean and green dashed line indicates on-treatment mean. Differences in means (± SEM) for each dependent variable grouped by time on treatment were determined by one-way ANOVA and *P* values for multiple comparisons with baseline were adjusted by Dunnett's *post*-*hoc* test (marked by letter “a”) and by the method of Benjamini, Krieger and Yekutieli [36] for false discovery rate (FDR) (*) where *P* ≤ 0.05. **e** Correlation analysis of Treg activity determined by area under the curve (AUC) versus UPDRS, Parts II + III. **f** Correlation analysis of Treg activity as determined by 50% Inhibitory Treg number versus UPDRS, Parts II + III. For both correlation analyses, regression bands are indicated by dashes lines encompassing the 95% confidence intervals (red) and the 95% prediction values (blue). Pearson values are denoted on each graph and were determined using Pearson product-moment correlation coefficients. Best-fit lines were determined by linear regression

Evaluation of peripherally isolated CD4^+^CD25^+^ Tregs revealed a significant increase in immunosuppressive capacity following initiation of sargramostim at all times measured during treatment and at 1 month after drug cessation (Fig. 2a, b). The mean Treg-induced inhibition at each sampling time was determined as area under the curve (AUC) as a function of Treg number and as the number of Treg cells necessary to achieve 50% inhibition of T cell proliferation, as previously described [28]. Quantification of Treg activity as the AUC revealed a significant elevation in function at all time points during sargramostim treatment compared to the mean AUC at the pretreatment baseline (Fig. 2c). In addition, determination of Treg activity as the number of Tregs necessary for 50% proliferation inhibition indicated that under the sargramostim treatment the Treg population had 75% greater mean capacity to significantly inhibit proliferation of CD4^+^CD25^−^ T responders (Tresp) compared to Treg isolates tested before sargramostim treatment (Fig. 2d). The increased Treg activity was maintained for at least 1 month after treatment cessation. Together, these data confirm increased Treg function at all sampling times throughout the study. Furthermore, correlation analyses of Treg activity with combined UPDRS Parts II and III motor scores indicated that the increased motor scores correlate with decreased Treg activity measured either as AUC (Fig. 2e) or as the number of Tregs necessary to attain 50% inhibition of T cell proliferation (Fig. 2f).

**Fig. 3:**
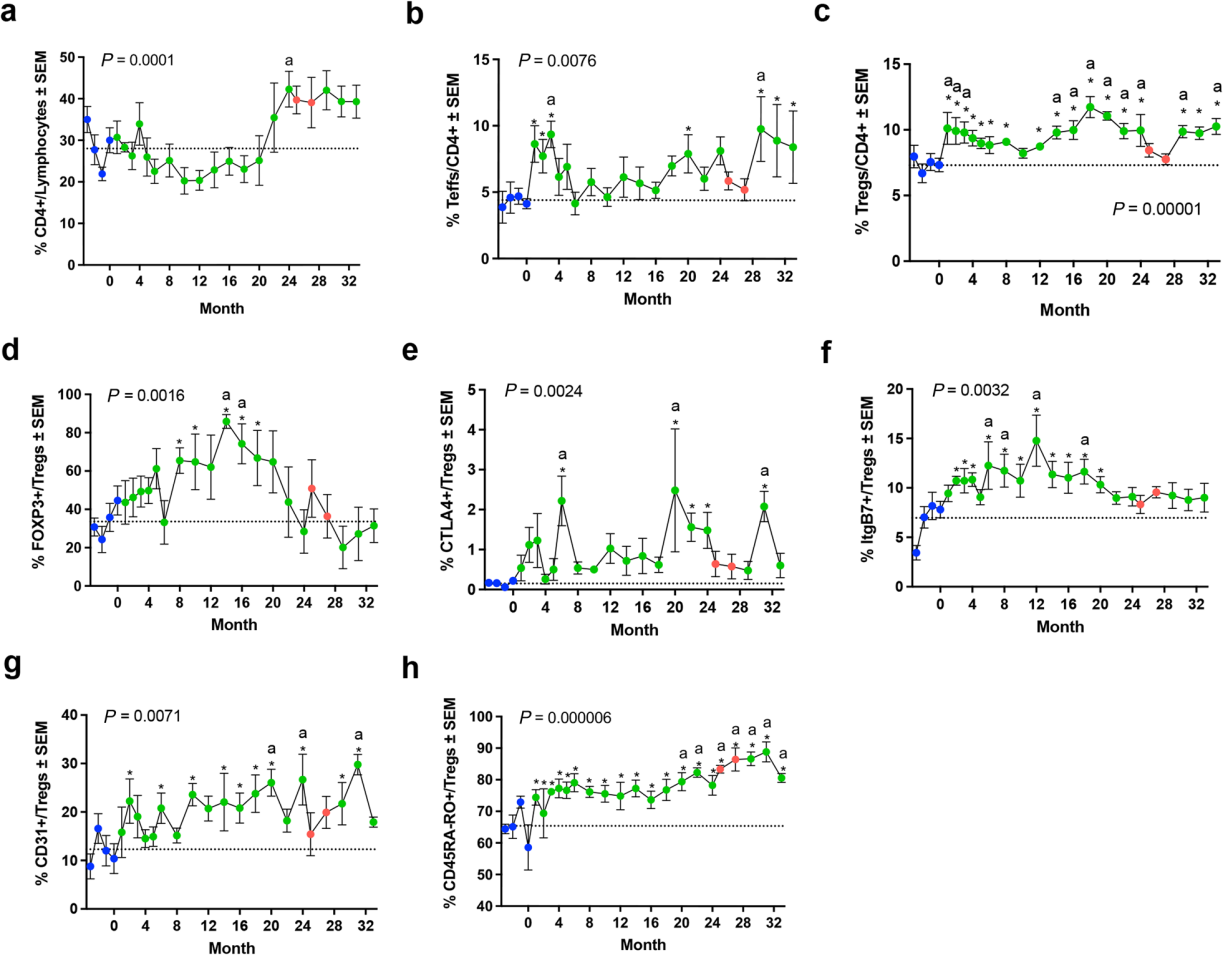
Sargramostim treatment stabilizes immunosuppressive surface markers of regulatory T cells. Quantification of **a** CD4^+^ lymphocytes, **b** CD4^+^ CD127highCD25^+^ Teffs, **c** CD4^+^ CD127lowCD25^+^ Tregs, **d** FOXP3^+^ Tregs, **e** CTLA^+^ Tregs, **f** ItgB7^+^ Tregs, **g** CD31^+^  Tregs, and **h** CD45RA-CD45RO^+^ Tregs over the course of treatment. Variables were measured at baseline (blue nodes), during drug treatment (green nodes), and during drug intermission (red nodes). Blue dashed lines indicate mean baseline measurement. Differences in means (± SEM) for each dependent variable grouped by time on treatment were determined by one-way ANOVA, and *P* values for multiple comparisons with baseline were adjusted by Dunnett's *post-hoc* test (marked by letter “a”) and by the method of Benjamini, Krieger and Yekutieli [36] for false discovery rate (FDR) (*) where* P* ≤ 0.05

Long-term treatment with sargramostim resulted in a non-significant elevation in CD4^+^ T lymphocytes and CD4^+^CD25^+^CD127-high Teff during the first 24 months of treatment (Fig. 3a, b). Following drug cessation, re-introduction of sargramostim resulted in a significant increase in both populations. Additionally, CD4^+^CD25^+^CD127-low Treg frequencies were significantly increased throughout 24 months of treatment (Fig. 3c). Following drug cessation, Treg levels returned to baseline, but upon re-introduction of drug, Treg levels were again significantly elevated within 2 months. Flow cytometric evaluation of Treg immunosuppressive and migratory markers also revealed sustained increases in FOXP3+ , CTLA+ , ItgB7+ , CD31+ and CD45RO+ Treg populations over time (Fig. 3d–h). The increased Treg markers paralleled Treg immunosuppressive data, indicating a population of Tregs with higher overall function (Fig. 2a–d), which may be due in part to increases in Treg subset frequencies, suppressive capabilities, or both.

### Effect of anti-sargramostim antibodies on Treg number and function, and UPDRS scores

**Fig. 4:**
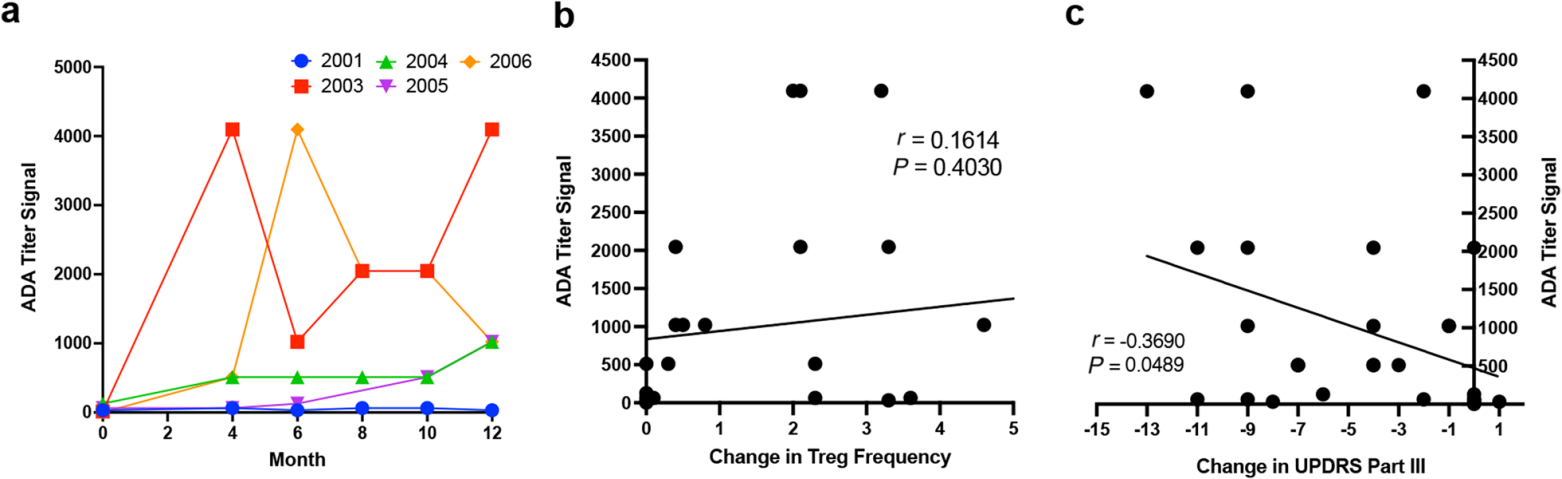
Presence of anti-drug antibodies (ADA) does not negatively affect Treg populations or UPDRS motor scores. **a** Anti-sargramostim neutralizing serum antibody titers collected from peripheral blood of individual subjects. **b** Correlation of ADA titers with change in Treg frequency for all subjects. **c** Correlation of ADA titers with change in UPDRS Part III scores for all subjects. Correlations were determined using Pearson product-moment correlation coefficients, and *P*-values determined for correlation coefficients greater than 0.25. Best-fit lines were determined using linear regression

Presence of neutralizing anti-sargramostim antibodies was evaluated in serum isolated from peripheral blood of individual subjects before and during the first 12 months of treatment. Four of the initial 5 subjects developed anti-drug antibodies (ADAs) within the first 4 months of treatment (Fig. 4a). However, correlation analyses revealed that the increased Treg numbers and the improved motor function were not stunted by or directly correlated with the presence of neutralizing antibodies (Fig. 4b, c). This affirmed our earlier clinical trial indicating that the anti-sargramostim antibodies produced little or no adverse effects on UPDRS scores or Treg function [26]. Previously, significant titers of neutralizing antibodies were present at times during sargramostim treatment when UPDRS Part III scores were below pretreatment levels, and Treg frequencies and function were significantly increased compared to placebo controls.

### Peripheral monocyte autophagy profiles

**Fig. 5:**
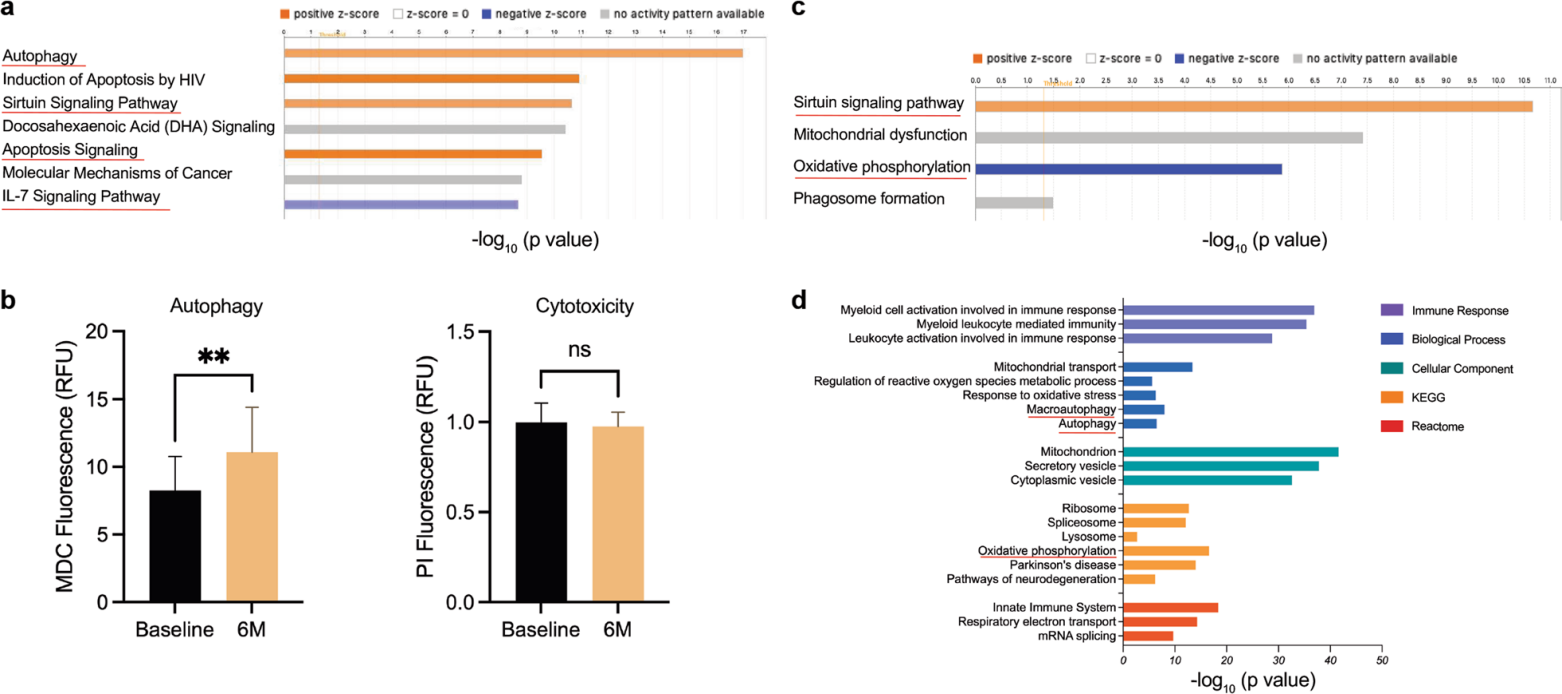
Autophagy is significantly increased in monocytes after 6 months of sargramostim treatment. **a** Canonical pathway enrichment analysis of 84 key autophagy gene measurements was performed using Ingenuity Pathway Analysis (IPA; Qiagen). Orange color (activation), blue color (inhibition), and grey color (no activity pattern). **b** Monodansylcadaverine (MDC) and propidium iodide (PI) fluorescence indicating autophagy and cytotoxicity, respectively, in monocytes at 6 months of sargramostim treatment compared to baseline (*n* = 4). Data are represented as mean ± SD. Statistical significance between the groups was determined with paired Student’s *t*-test and *P* ≤ 0.05 was considered as statistically significant. **c** Canonical pathway enrichment analysis of differentially expressed proteins in monocytes at 6 months of sargramostim treatment using IPA (Qiagen). Orange color (activation), blue color (inhibition), and grey color (no activity pattern) (figure modified from previous publication [33]). **d** Gene ontology (GO)-term functional enrichment by five categories (immune response, biological process, cellular component, KEGG, and Reactome) was performed using Cytoscape in conjunction with the plug-in ClueGO and in consideration of a prior published report [25]. 6 M: 6 months; *RFU* Relative fluorescence units, *KEGG* Kyoto encyclopedia of genes and genomes

In mechanistic studies, sargramostim therapy was evaluated for its effects on peripheral blood monocyte function on α-syn evolution to fibrillary aggregates during 6 months of treatment. Autophagy was a focus in this study as it represents a principal intracellular proteolytic process for clearance of α-syn aggregates [37]. Moreover, previous studies have demonstrated clear associations of α-syn aggregates with the onset and progression of PD [8, 12, 16, 18]. Moreover, monocytes were studied as they represent the source of perivascular brain macrophages and microglia. To this end, we investigated whether key genes involved in the autophagy pathways were affected during therapy (Additional file 2). However, while no significant alterations in the expression of singular screened genes were delineated, which may potentially be due to the small sample size, genetic variation between subjects, and/or small number of screened genes, functional and pathway enrichment analyses of autophagy-regulated genes in monocytes showed significant alterations before versus following treatment. IPA evaluations demonstrated that autophagy (*P* = 1 × 10^−17^) and sirtuin signaling pathway (*P* = 2.51 × 10^−11^) were significantly upregulated following initiation of sargramostim treatment (Fig. 5a and Additional file 2). Moreover, IPA also showed enrichment of apoptosis and immune signaling pathways (Fig. 5a and Additional file 2). Taken together, these results highlight potential disease-altering pathways affected within the monocyte populations. Additionally, PPI network(s) linked to restoration of tissue homeostasis were uncovered through biological, cellular, molecular, genetic, and reactome analyses. These interactions were further supported by functional protein association network using STRING analysis, which demonstrated associations between autophagy, immune, and apoptosis pathways (Additional file 2). The biological process analysis confirmed enrichment of autophagy events (*P* = 9.76 × 10^−50^) as well as linkages of macroautophagy (*P* = 5.31 × 10^−33^), autophagy regulation (*P* = 1.14 × 10^−23^), autophagosome assembly (*P* = 5.47 × 10^−26^), and autophagy to the mitochondrion (*P* = 5.19 × 10^−23^) (Additional file 2). Moreover, cellular component analysis showed enrichment of key autophagy subcellular structures including the autophagosome (*P* = 3.40 × 10^−33^), autolysosome (*P* = 2.10 × 10^−7^), and phagocytic vesicle (*P* = 8.08 × 10^−6^) (Additional file 2). To confirm the transcriptomic and proteomic analyses, MDC was used as a specific fluorescent marker to quantitate autophagic vacuoles [38]. MDC-stained monocytes from subjects treated with sargramostim showed a significant 34% increase in fluorescent intensity compared to cells at baseline (*P* = 0.003), indicating increases in autophagic vacuole formation (Fig. 5b). No evidence of differential monocytic cytotoxicity was observed between treated and baseline samples. Additionally, as reported previously, modulations of sirtuin signaling, oxidative phosphorylation, and phagosome formation were noted following sargramostim treatment (Fig. 5c, d) [33]. These data together suggest that sargramostim increases autophagic structures or function as key processes in maintaining homeostasis and may be linked to removal of misfolded proteins such as α-syn that accumulate during PD [37]. The results demonstrate “putative” protective mechanisms of sargramostim recorded during the early treatment.

## Discussion

Standard approaches for PD treatment center on dopamine replacement using either carbidopa/levodopa, dopamine agonists, or agents that prolong the actions of endogenous dopamine [2]. Additionally, deep brain stimulation surgery is utilized when anti-parkinsonian medication responses can no longer affect disease signs and symptoms [39]. Alternative medicine and integrative medicine approaches can improve the sense of well-being and overall health. Exercise, diet, and behavioral interventions have also proven to be beneficial, leading to improvements in the quality-of-life [40–42]. Previously, we have shown that sargramostim stimulates peripheral T cell and monocyte responses in vivo that affect reactive oxygen species, autophagy, and anti-inflammatory responses and is linked to enhanced motor activity and function associated with beneficial outcomes [26, 28, 33]. Data provided in the current report support this notion by clear demonstration of sustained effects on immune function that occur in a safe and well-tolerated therapeutic setting.

In the current report, an extended therapeutic regimen of sargramostim was demonstrated to be safe for 33 months following drug administration in PD subjects. Expected adverse events included increased WBC, injection-site reactions, and bone and chest pain that have been previously reported with sargramostim treatment [19, 26, 28]. Hematologic and metabolic profiles were within normal limits during treatment, and immune and motor functions improved during a 5-day-on and 2-day-off treatment regimen. Potential therapeutic response was highlighted with UPDRS scores returning to baseline during 3 months of drug discontinuation. These were then restored after treatment re-initiation. The findings reflect a stable symptomatic response. This study also supports a body of accumulating research highlighting a prominent role of the innate and adaptive immune systems in both development and progression of PD along with other nervous system pathologies [7, 43–47]. Specifically, a body of pre-clinical and translational studies demonstrate that Teff responses affect disease onset and progression by exacerbating innate microglial inflammation [48]. In contrast, Tregs have been shown to suppress adaptive and innate effector populations [10]. Such results have been demonstrated in diverse neurodegenerative disorders including multiple sclerosis, Guillain–Barre syndrome, neuropathic pain, traumatic brain injuries, stroke, amyotrophic lateral sclerosis, PD, and AD [28, 49–58].

Tregs hold significant promise as candidates to affect immune transformation and develop novel therapeutics in multiple clinical settings. For instance, in animal models, transient depletion of Tregs was demonstrated to facilitate AD cognitive decline and be linked to diminished microglial clearance of amyloid [55]. On the other hand, restoration of Treg numbers and function increased microglial plaque clearance and improved cognitive functions. Additionally, reductions of Treg numbers and suppressive function paralleled AD clinical progression, while following Treg expansion, cell function was restored including control of pro-inflammatory macrophage activities. Each support the notion that restoration of Treg function can serve to restore brain homeostasis through reductions in the inflammatory disease state. Parallel findings have been observed in a spectrum of autoimmune and degenerative diseases of the nervous system where disease severity was found to be associated more with changes in T cell numbers and function than with age, onset, duration, and/or progression [7, 43–47]. Taken together, each of these findings supports the importance of transforming Treg function, as supported in the current report, in controlling immune responses and demonstrates a sustained multiyear neuroprotective strategy to halt disease progression and maintain homeostatic control.

**Fig. 6:**
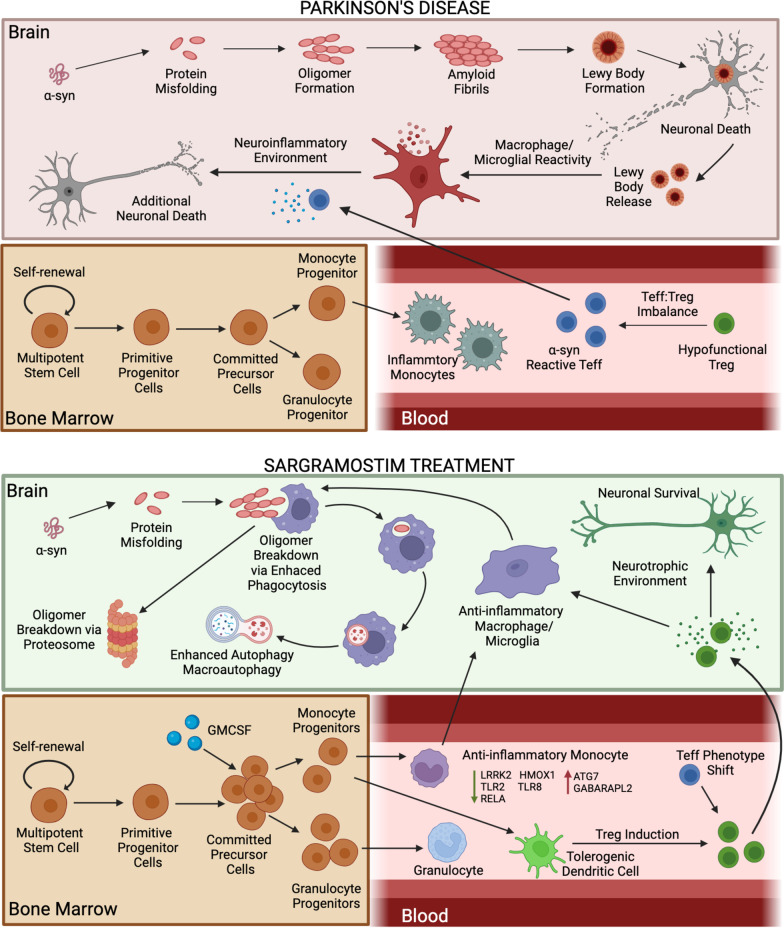
Proposed therapeutic mechanism for sargramostim. During PD progression, native alpha-synuclein (α-syn) becomes modified and misfolded. Modification results in formation of oligomers that aggregate into fibrils due to dysfunctional protein clearance and breakdown. Fibrils coalesce into intra- and extraneuronal inclusion bodies (Lewy bodies) resulting in dopaminergic neuronal cell death. Dead and dying neurons release Lewy bodies and aggregated α-syn into the extracellular environment that is taken up by resident microglia and infiltrating macrophages, causing the initiation of an pro-inflammatory signaling and reactive phenotype. Reactive microglia/macrophages secrete neurotoxic mediators in response to misfolded protein, resulting in additional neuronal death. The imbalance of inflammatory monocytes and T effector cells (Teff) with anti-inflammatory regulatory T cells (Treg) contributes to the peripheral inflammatory milieu associated with disease. To suppress this response, peripheral administration of sargramostim (GM-CSF, Leukine) results in proliferation of myeloid progenitor cells within the bone marrow that mobilize to the bloodstream following maturation into anti-inflammatory monocytes, granulocytes, and tolerogenic dendritic cells. Transcriptomic and proteomic evaluations of circulating monocytes after treatment with sargramostim revealed a monocyte phenotype with increased expression of CD93, CD163, ATG7, and GABARAPL2, and decreased expression of LRRK2, HMOX1, TLR2, TLR8, and RELA, indicating increased antioxidant, anti-inflammatory, and autophagic functions. Additionally, sargramostim treatment results in induction of immunosuppressive Tregs. Resulting tolerogenic dendritic cell-induced Treg populations show elevated FOXP3, CTLA-4, ITGB7, CD45RO, and CD31, which support a stable immunosuppressive phenotype with enhanced migratory functions. Within the brain, infiltrating monocytes and microglia become polarized into an anti-inflammatory phenotype with enhanced phagocytosis, autophagy, and macroautophagy. This leads to increased protein clearance, proper oligomer breakdown, decreased Lewy body formation, restoration of a homeostatic microenvironment, and ultimately, decreased neuroinflammation and neurodegeneration. Additionally, infiltration of induced immunosuppressive Treg to the sites of inflammation enhances an anti-inflammatory microglial phenotype and control of neural homeostasis, which further contributes to a disease-modifying neuroprotective environment

Tregs serve as a subpopulation of immunosuppressive T cells which sustain immune homeostasis. This is under the control of a Treg-specific, master-regulating transcription factor, FOXP3, by maintaining self-tolerance. Tregs serve as negative regulators of inflammation during autoimmune disease. For neurodegenerative diseases, Tregs can affect nervous system pathologies [59–61]. Tregs are reduced in number and function including their ability to suppress activated pro-inflammatory macrophages. When function is restored, Tregs display increased expression of factors such as FOXP3, IL2Ra (CD25), NT5E (CD73), IL10, IL13, CTLA4, PDCD1 (PD1), and GRZMB [62]. There is a shift towards a pro-inflammatory peripheral immune response in PD with the loss of Treg suppressive functions, affecting disease progression typified by a systemic pro-inflammatory response [26, 28, 35]. The restoration and enhancement of Treg suppressive functions in this study underlies the importance of control over adaptive immune activities as a therapeutic approach for PD [63]. Long-term sargramostim therapy resulted in sustained increases in peripheral Tregs that display an immunosuppressive phenotype. They migrate to sites of disease where they perform anti-inflammatory functions (Fig. 6). Additionally, presence of this population was correlated with decreases in UPDRS Part III scores, indicating their potential role in affecting disease course. Lastly, although neutralizing ADAs were developed in some subjects, they are not likely to have a detrimental effect as higher ADA titers were not directly correlated with Treg numbers and function or decreased motor score improvements. However, these evaluations were done only at 12 months post-drug initiation.

Apart from inducing adaptive immune response alterations, GM-CSF has also been shown to enhance phagocytic function in innate immune populations such as autophagy. Autophagy is a degradation process to remove defective cellular components within the cell. There are three different classes of autophagy in mammalian cells, chaperone-mediated autophagy, macroautophagy, and microautophagy [64]. Autophagy helps to maintain cellular homeostasis through intracellular effective turnover of proteins and damaged organelles [65]. Impaired autophagy and mitophagy are observed in brain regions of PD patients, and autophagic degeneration is seen in dopaminergic neurons of the substantia nigra from PD patients [66]. Herein, bioinformatic analysis of key autophagy genes and autophagic vacuole formation showed that sargramostim—in the early stage of treatment—enhanced autophagy and sirtuin signaling pathways that may be linked to clearance of misfolded and aggregated proteins. These data support recent works demonstrating transcriptomic and proteomic signatures during sargramostim treatment that suggest a shift from an inflammatory neurotoxic to an anti-inflammatory and neuroprotective environment [33]. These included antioxidant, anti-inflammatory, and autophagy activities in PD monocytes recorded after initiation of sargramostim treatment. We hypothesize that enhanced autophagy recorded during the early treatment stage has the potential to affect neuronal survival and lead to neuroprotective outcomes (Fig. 6). This would occur through enhanced α-syn breakdown and subsequent removal, with Treg-induced decreases in neuroinflammation.

While GM-CSF-sargramostim is a known myeloid growth factor affecting functions of multiple mononuclear phagocytes (monocytes, macrophages, microglia and dendritic cells), it possesses a pleotropic effect on immunity. Indeed, while most PD therapies are designed to target dopamine pathways, sargramostim may protect the brain indirectly through its effects on peripheral hematopoiesis, innate and adaptive immunity, and metabolism. Each avenue may lead to neuroprotective outcomes. For example, in stroke and traumatic brains as well as spinal cord injury animal models, GM-CSF has neuroprotective and anti-apoptotic activities, enhances cerebral blood flow, decreases lesion formation, and restores locomotor function [67–72]. In a range of neurodegenerative disease models, GM-CSF treatment is linked to improved locomotor function and cognitive function that correlate to altered innate and adaptive immune functions [21, 22, 24, 25, 73–75]. Additionally, sargramostim can readily cross the blood–brain barrier and may further improve disease outcomes through direct effects on the nigrostriatal pathway via receptor binding [76]. In animal studies, GM-CSF directly infused into the brain causes increased levels of neurotrophins and neurotransmitters that include serotonin and norepinephrine [24, 77]. However, GM-CSF treatment has not been shown to affect the levels of dopamine and its metabolites, such as 3,4-dihydroxyphenylacetic acid. Therefore, the potential neuroprotective effect of sargramostim in these models is not believed to be due to increased dopamine production or dopamine signaling.

## Study limitations

The current study was designed as a small, open-label investigation seeking to evaluate the safety and tolerability of sargramostim for an extended time. While there was no placebo control, the incorporation of subject baseline evaluations allowed for treatment comparisons. The study evaluated a small number of PD subjects early in their disease course. Therefore, this is not an evaluation of early, mid, and late diseases. Additional factors that may limit the interpretation of the data sets include disease-required anti-Parkinsonian medications. The unblinded evaluations and lack of UPDRS motor assessments in both “on” and “off” states are other limitations. Although statistically significant results were identified, the motor and neurological improvements and biomarker evaluations require validation in a Phase II double-blind, placebo-controlled study to be tested for clinical efficacy.

## Conclusions

In the current open-label study, sargramostim treatment led to stable UPDRS Part II and III scores. The improved scores were observed 3 months following treatment and were sustained during the study’s course. Additionally, the genes and proteins found affected by sargramostim were not related to dopamine production or neurotransmission but linked to autophagy, neuroinflammation, and neuroprotection [33]. The findings were recorded in a small number of subjects. Therefore, larger clinical studies are required to confirm whether sargramostim is associated with improved clinical outcomes.

## Supplementary Information

**Additional file 1. Figure S1.** Individual UPDRS Part II scores over time. **Figure S2.** Individual UPDRS Part III scores over time. **Table S1.** Complete blood count before and after initiation, pause, and restart of sargramostim treatment. **Table S2.** Comprehensive metabolic panel before and after initiation, pause, and restart of sargramostim treatment. **Table S3.** T cell panel before and after initiation of sargramostim treatment.

**Additional file 2.** Genes regulated by sargramostim treatment.

## Data Availability

The datasets generated for this study and full study protocol are available upon request from the corresponding author.

## Abbreviations

α-syn: Alpha-synuclein
AD: Alzheimer’s disease
ADA: Anti-drug antibody
AUC: Area under the curve
FBS: Fetal bovine serum
FOXP3: Forkhead box P3
GM-CSF: Granulocyte–macrophage colony-stimulating factor
IPA: Ingenuity pathway analysis
MDC: Monodansylcadaverine
PD: Parkinson’s disease
PPI: Protein–protein interaction
STRING: Search Tool for the Retrieval of Interacting Genes/Proteins
Teff: Effector T cell
Treg: Regulatory T cell
UPDRS: Unified Parkinson’s Disease Rating Scale
