# Supplementary material for: An open-label multiyear study of sargramostim-treated Parkinson’s disease patients examining drug safety, tolerability, and immune biomarkers from limited case numbers
Source: Transl Neurodegener. 2023 May 22;12:26. doi: 10.1186/s40035-023-00361-1 (PMC10201023; doi:10.1186/s40035-023-00361-1)

**Additional file 1**

**
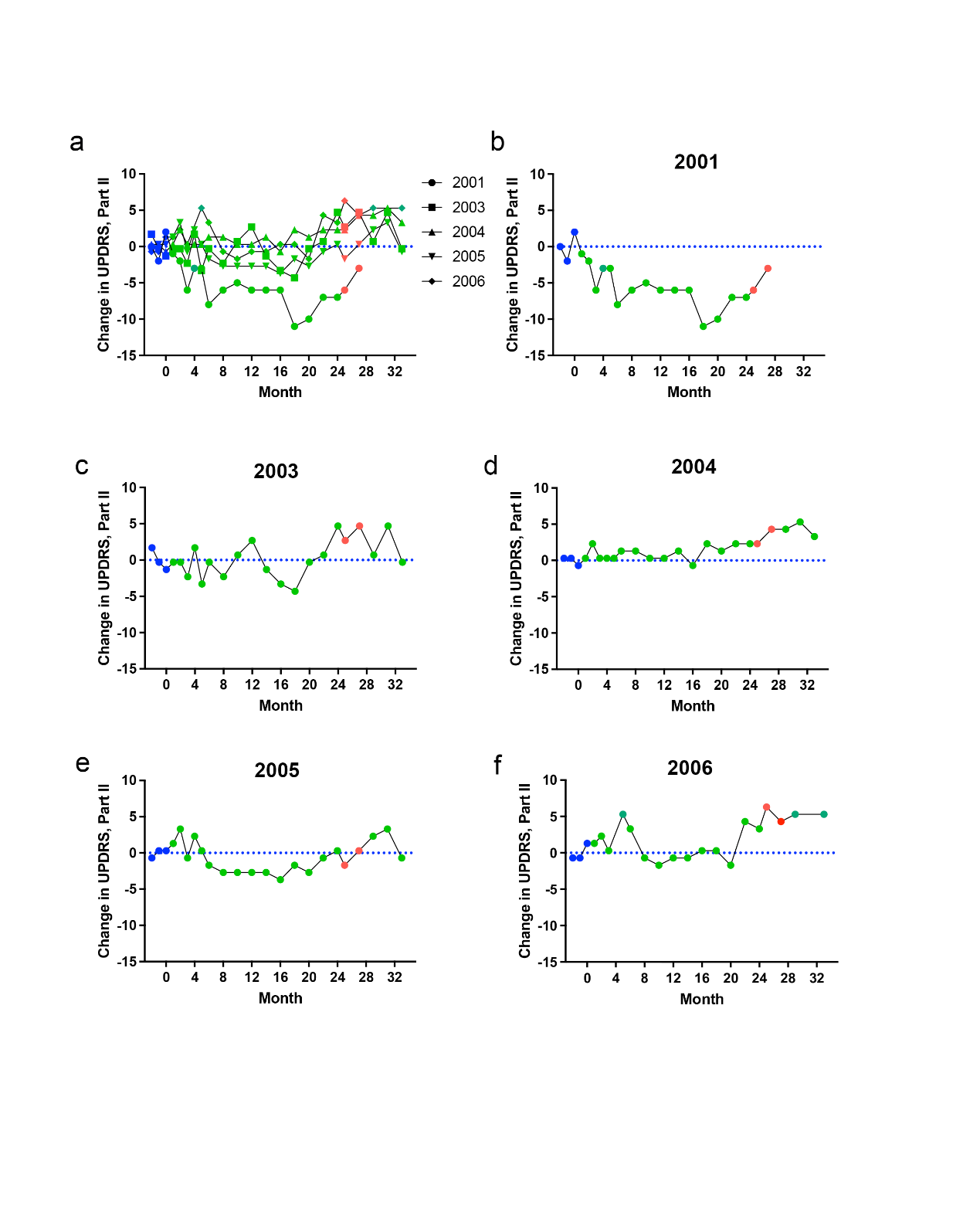
**

**Figure S1.** *Individual UPDRS Part II scores over time.* **(a)** Change from baseline in UPDRS Parts II scores for all subjects. Change from baseline in UPDRS Part II scores for subjects 2001 **(b),** 2003 **(c),** 2004 **(d),** 2005 **(e),** and 2006 **(f)** separately. Blue nodes indicate baseline evaluations. Blue dashed line indicates baseline average. Green nodes indicate “on” sargramostim treatment and red nodes indicate drug cessation.


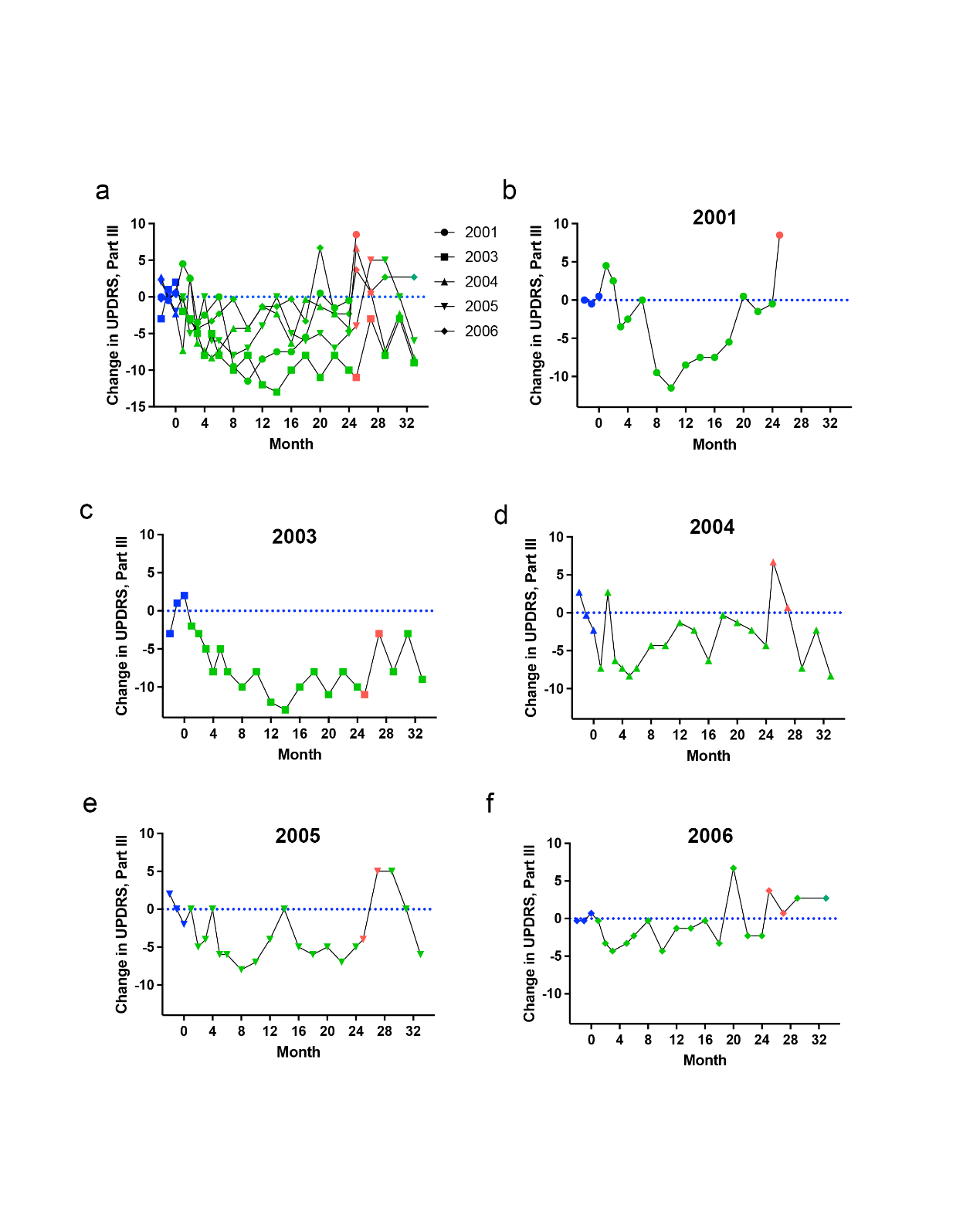


**Figure S2.** *Individual UPDRS Part III scores over time.* **(a)** Change from baseline in UPDRS Parts III scores for all subjects. Change from baseline in UPDRS Part III scores for subjects 2001 **(b),** 2003 **(c),** 2004 **(d),** 2005 **(e),** and 2006 **(f)** separately. Blue nodes indicate baseline evaluations. Blue dashed line indicates baseline average. Green nodes indicate “on” sargramostim treatment and red nodes indicate drug cessation.


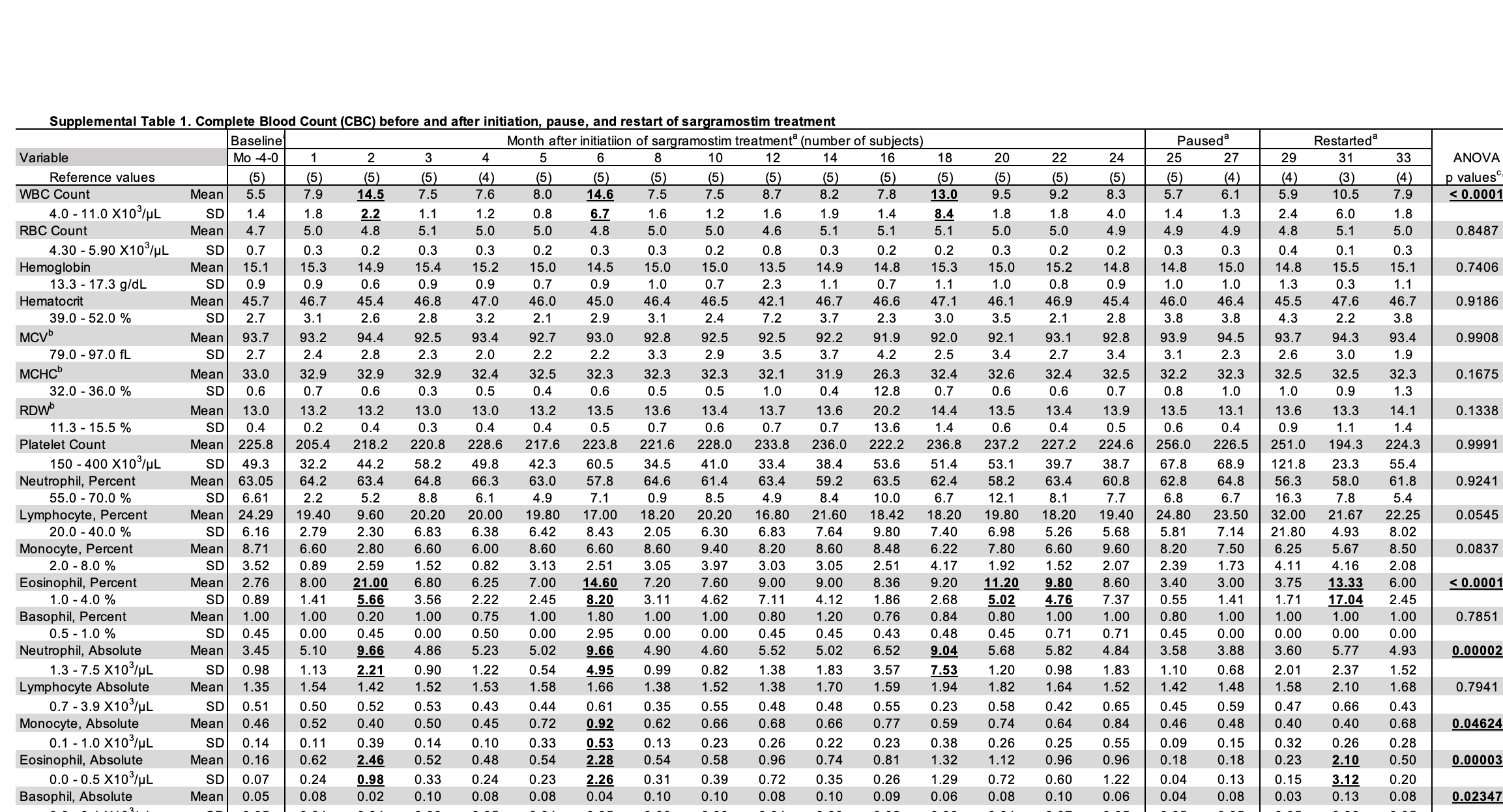


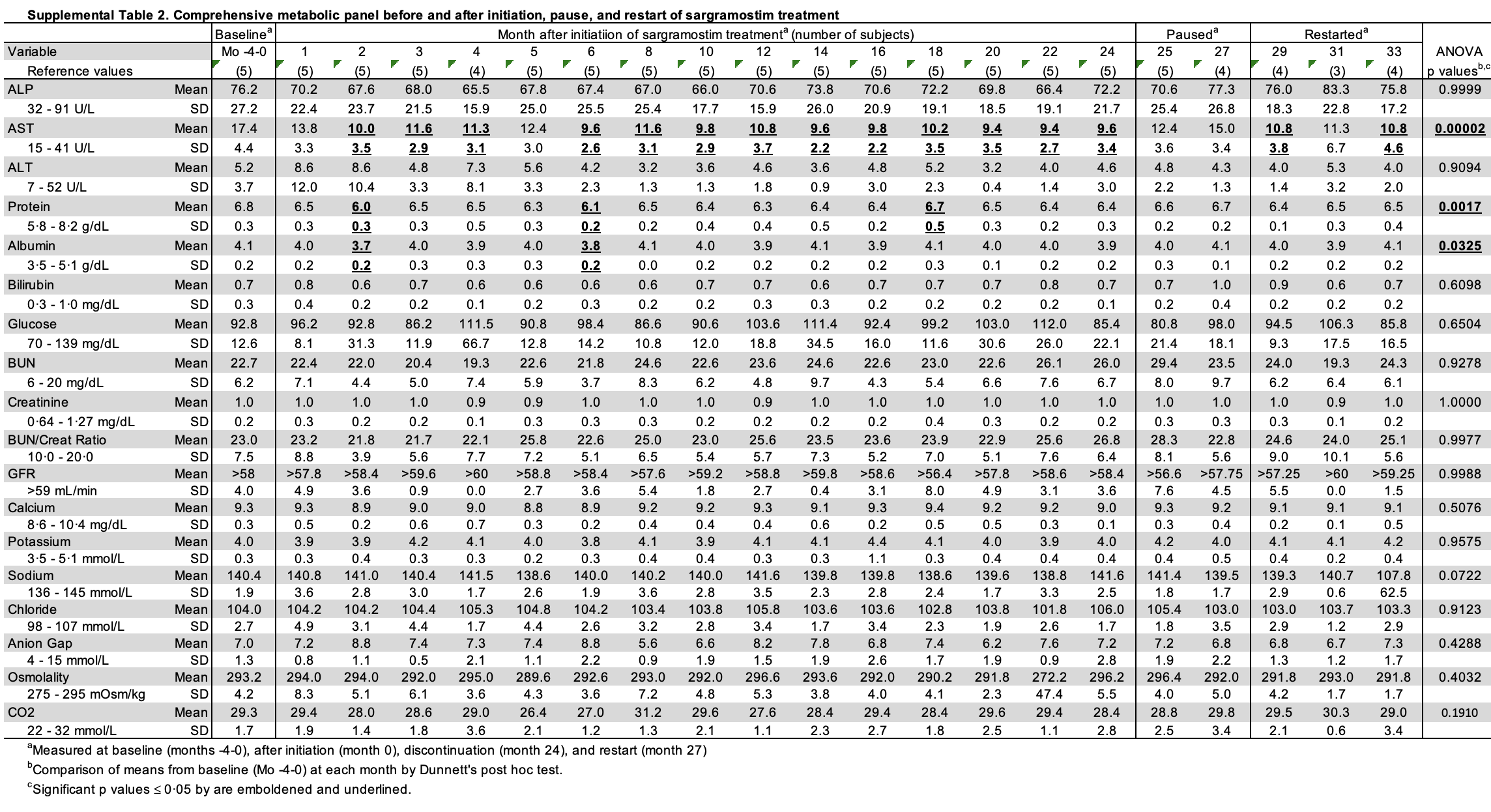


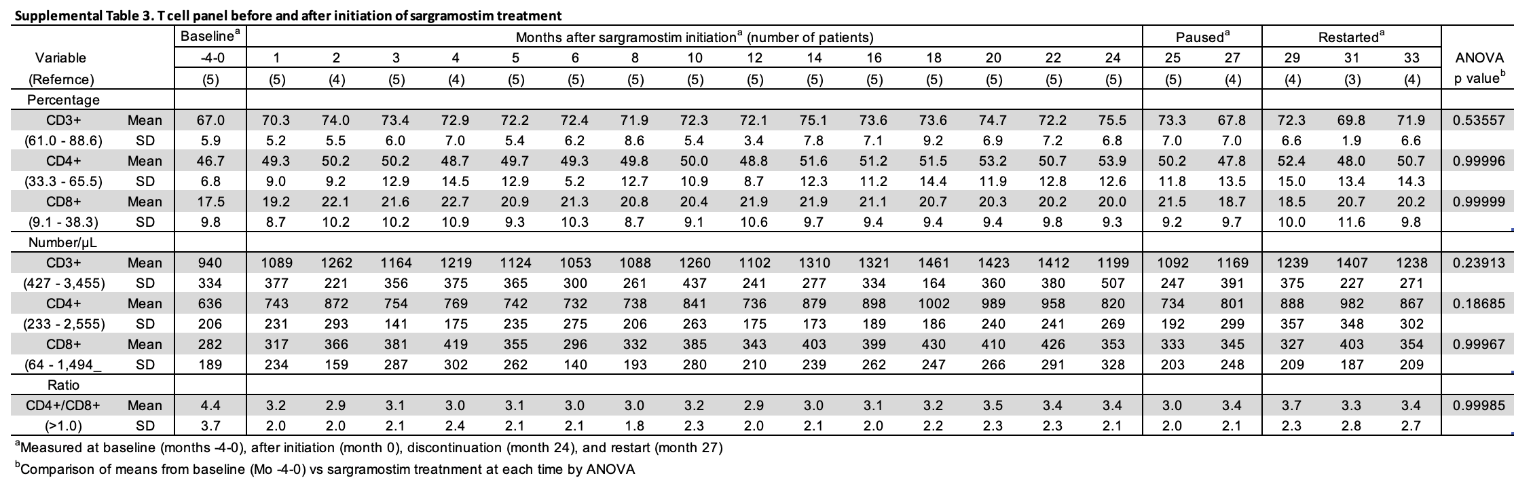

Supplement: Supplementary file 1 — Additional file 1. Figure S1. Individual UPDRS Part II scores over time. Figure S2. Individual UPDRS Part III scores over time. Table S1. Complete blood count before and after initiation, pause, and restart of sargramostim treatment. Table S2. Comprehensive metabolic panel before and after initiation, pause, and restart of sargramostim treatment. Table S3. T cell panel before and after initiation of sargramostim treatment. [file 40035_2023_361_MOESM1_ESM.docx]
